# A zingiberene–selenium nanoparticle formulation modulates oxidative, inflammatory, and osteogenic responses during femoral fracture healing in rats

**DOI:** 10.3389/fvets.2026.1908406

**Published:** 2026-10-02

**Authors:** Alaa Jameel A. Albarakati

**Affiliations:** Surgery Department, College of Medicine, Al-Qunfudah Branch, Umm Al-Qura University, Makkah, Saudi Arabia

**Keywords:** bone remodeling, femoral fracture, inflammation, oxidative stress, selenium nanoparticles, zingiberene

## Abstract

**Introduction:**

Bone fracture healing involves coordinated inflammatory, oxidative, and remodeling processes. This study evaluated free zingiberene and a zingiberene-selenium nanoparticle (zingiberene-SeNP) formulation in a rat femoral fracture model.

**Methods:**

Twenty-four adult male Wistar rats were assigned to intact control, fracture control, fracture plus zingiberene (10 mg/kg/day), or fracture plus zingiberene-SeNP formulation (0.5 mg/kg/day, expressed as total administered formulation mass) groups for 35 days. Outcomes included serum calcium, phosphate, magnesium, and alkaline phosphatase (ALP); malondialdehyde (MDA), nitric oxide (NO), reduced glutathione (GSH), superoxide dismutase (SOD), and catalase (CAT); C-reactive protein (CRP), cyclooxygenase-2 (COX-2), prostaglandin E₂ (PGE₂), myeloperoxidase (MPO), tumor necrosis factor-alpha (TNF-α), interleukin-1 beta (IL-1β), and nuclear factor-kappa B (NF-κB); C-terminal telopeptide of type I collagen (CTX-I), calcitonin, osteocalcin, osteoprotegerin (OPG), and bone morphogenetic protein-1 (BMP-1), together with radiographic and hematoxylin and eosin (H&E) histology.

**Results:**

Fracture disrupted mineral, oxidative, inflammatory, and bone-turnover profiles. Both treatments improved multiple biochemical and inflammatory indices versus fracture control. The zingiberene-SeNP formulation showed stronger effects than free zingiberene for selected endpoints, including MDA, GSH, ALP, PGE₂, TNF-α, IL-1β, NF-κB, CTX-I, calcitonin, osteocalcin, OPG, and BMP-1. Radiographic and histological findings were consistent with improved callus organization and bone maturation.

**Discussion:**

Overall, both treatments favorably modulated fracture-repair responses, with greater effects of the nanoformulation for selected parameters.

## Introduction

Bone fractures are a major clinical problem, and approximately 10–15% progress to delayed union or non-union, with substantial consequences for mobility and quality of life (1, 2). Fracture repair proceeds through overlapping inflammatory, reparative, and remodeling phases that require coordinated cellular activity, growth-factor signaling, extracellular-matrix turnover, and mineralization (3). Successful healing depends on a balanced inflammatory response, controlled oxidative stress, adequate mineral availability, and coordinated osteoblast and osteoclast activity. Autografts remain an important option for difficult fractures, but donor-site morbidity, limited graft volume, and procedure-related complications motivate the development of adjunctive therapies (4, 5).

Nanomedicine offers potential strategies to support bone repair through local or systemic delivery of bioactive compounds. Nanoparticles have a high surface-area-to-volume ratio and can modify compound stability, tissue distribution, and release characteristics (6, 7). However, these potential advantages depend on formulation-specific properties, including particle size, morphology, surface charge, composition, loading, release, and stability, which must be characterized before pharmacokinetic or bioavailability benefits can be inferred (8, 9).

Selenium is an essential trace element involved in antioxidant defense, immune regulation, and thyroid-hormone metabolism (10, 11). Selenium nanoparticles (SeNPs) have been investigated as alternatives to conventional selenium forms because their biological effects and toxicity profiles may differ according to size, surface chemistry, and dose (12, 13). Preclinical studies suggest that some selenium-containing nanomaterials can support osteogenic differentiation and fracture repair (14, 15). These intrinsic biological effects also mean that an SeNP-only control would be required to distinguish the contribution of selenium from that of a compound associated with the nanoparticles.

Zingiberene is a sesquiterpene hydrocarbon and a major constituent of ginger (*Zingiber officinale*) essential oil. Ginger-derived compounds have demonstrated antioxidant and anti-inflammatory activities in experimental systems (16–19). These effects include scavenging of reactive oxygen species, modulation of endogenous antioxidant enzymes, and suppression of inflammatory mediators such as NF-κB, TNF-α, IL-1β, COX-2, and PGE₂ (20–23). Nevertheless, evidence obtained with ginger extracts or other ginger constituents, such as gingerols, cannot be assumed to represent the specific activity of purified zingiberene.

Combining natural products with selenium nanoparticles may produce a formulation with biological effects relevant to fracture repair, but the separate contributions of the natural compound, selenium, and the nanoparticle system must be considered. In the present study, the available design compared free zingiberene with a zingiberene-SeNP formulation at non-equivalent administered doses and did not include an SeNP-only group. Accordingly, the study was designed as an exploratory comparison of treatment-associated response patterns rather than a definitive test of synergy, enhanced bioavailability, or component-specific mechanism.

This study evaluated the effects of free zingiberene and a zingiberene-SeNP formulation in a rat femoral fracture model. The objectives were to examine: (1) serum calcium, phosphate, and magnesium concentrations together with ALP activity; (2) peri-fracture oxidative and inflammatory indices; (3) selected bone-turnover markers and NF-κB, OPG, and BMP-1 mRNA expression; and (4) qualitative radiographic and H&E histological features of fracture repair.

## Materials and methods

### Experimental animals and ethical approval

The experimental protocol was approved by the Biomedical Research Ethics Committee of Umm Al-Qura University (approval no. HAPO-02-K-012-2025-12-3126). Twenty-four adult male Wistar rats (5 months old; 150 ± 10 g) were obtained from King Abdulaziz University. Animals were housed under a 12-h light/dark cycle at 22 ± 2 °C and 50–60% relative humidity, with free access to standard laboratory chow and water. A one-week acclimatization period preceded the experimental procedures.

### Preparation of the zingiberene–selenium nanoparticle formulation zingiberene source

Zingiberene (purity 98%; CAS no. 495–60-3) was obtained from Sigma-Aldrich (St. Louis, MO, United States) (Figure 1).

**Figure 1:**
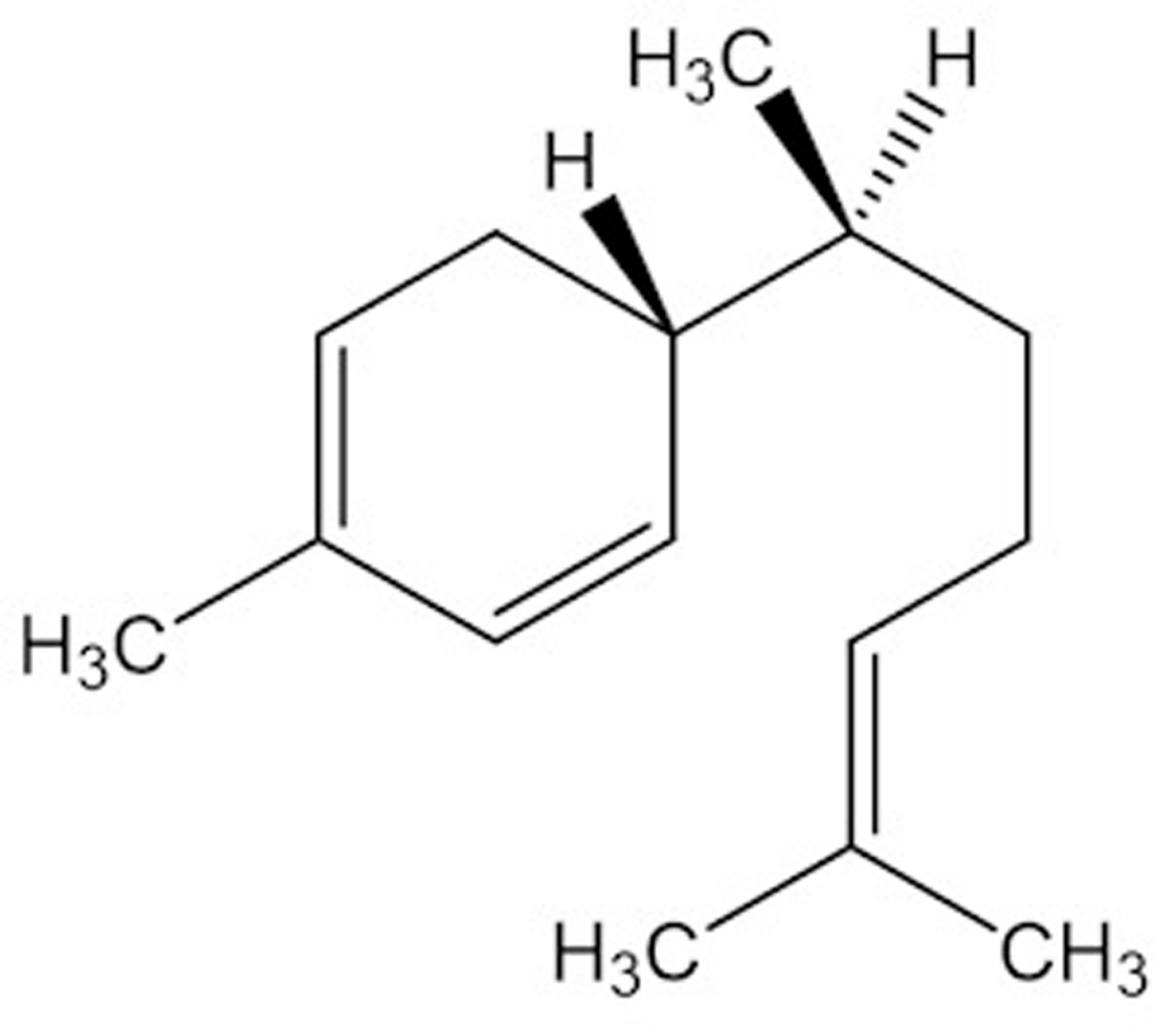
Chemical structure of zingiberene.

### Preparation and available physicochemical characterization

A 0.1 M aqueous sodium selenite (Na₂SeO₃) solution was mixed with a zingiberene solution. Ascorbic acid (1% v/v) was then added dropwise, and the mixture was stirred at room temperature for 24 h. Development of a red-orange color was considered consistent with elemental selenium formation. The prepared zingiberene-SeNP formulation was assessed by dynamic light scattering (DLS) for hydrodynamic particle-size distribution and polydispersity index (PDI), and by zeta-potential analysis for surface charge.

### Experimental design and fracture induction

Animals were randomly allocated to four experimental groups (*n* = 6 per group):

- Group 1 (Control): intact rats without fracture induction.
- Group 2 (Fracture control, FX): femoral fracture without therapeutic intervention.
- Group 3 (FX + zingiberene): femoral fracture with oral zingiberene (10 mg/kg/day).
- Group 4 (FX + zingiberene-SeNPs): femoral fracture with the prepared zingiberene-SeNP formulation (0.5 mg/kg/day, expressed as the total administered formulation mass).

The group size was selected based on sample sizes used in comparable published experimental fracture-healing studies (24, 25). The amount of peri-fracture muscle/soft tissue obtained from each animal was sufficient for the planned biochemical measurements. A standardized closed femoral diaphyseal fracture was induced in Groups 2–4 under sevoflurane anesthesia (Sevorane®). A three-point bending apparatus was used under fluoroscopic guidance to produce a transverse fracture at the junction of the proximal one-third and distal two-thirds of the femoral diaphysis. Immediate radiography was used to confirm a single, well-defined transverse fracture without comminution or intra-articular extension, consistent with the criteria described by Histing et al. (26). No internal or external fixation was applied. Because fracture alignment, rotation, and shortening were not graded using a standardized score, the unfixed model may have introduced inter-animal variability. Post-fracture analgesia was provided with buprenorphine (0.03 mg/kg, intramuscularly) twice daily for 5 days. Animals were monitored daily for their general condition, gait and use of the affected limb, and signs of pain or distress. Immediate post-fracture radiographs were used to verify acceptable fracture morphology as described above. No animals were excluded following fracture induction, and all 24 animals completed the experimental period.

Treatment began on day 1 after fracture induction and continued once daily by oral gavage for 35 consecutive days. Animals were allowed unrestricted cage activity and weight-bearing on the fractured limb throughout the study.

### Radiographic evaluation

Radiographs were obtained immediately after fracture induction (day 0) and at the final time point (day 35). Rats were anesthetized with sevoflurane and positioned prone for standardized anteroposterior imaging of the fractured femur. Images were first assessed descriptively for fracture-line visibility, callus formation, bone continuity, and cortical remodeling. In addition, the representative day-35 fracture radiographs were retrospectively classified using the Lane–Sandhu radiographic healing score (0–12 points) (27, 28). The scoring criteria: callus formation (0–4 points), fracture-line disappearance (0–4 points), and remodeling (0, 2, or 4 points)—are summarized in Table 1, with higher totals indicating more advanced radiographic healing. Panel A served as the immediate post-fracture day-0 reference, and panel B represented the intact control; neither was included in the day-35 fracture-healing score. Because only representative archived radiographs were available and the complete animal-level image set and original blinding records were unavailable, the Lane–Sandhu scores were used as descriptive representative-panel classifications only and were not subjected to inferential statistics.

**Table 1:** Radiographic and histological scoring criteria used for retrospective descriptive assessment of fracture healing.

Scoring system	Component/stage	Criterion	Points
Lane–Sandhu	Callus formation	No callus; callus occupying 25, 50, 75%, or 100% of the fracture/defect region	0, 1, 2, 3, 4
Lane–Sandhu	Fracture line	Clear; relatively clear; partial; basically vanished; completely vanished	0, 1, 2, 3, 4
Lane–Sandhu	Remodeling	No remodeling; remodeling of the intramedullary channel; full remodeling of cortex	0, 2, 4
Lane–Sandhu	Total score	Sum of callus formation, fracture-line, and remodeling components	0–12
Huo	Stage 1	Fibrous tissue	1
Huo	Stage 2	Predominantly fibrous tissue with a small amount of cartilage	2
Huo	Stage 3	Equal mixture of fibrous and cartilaginous tissue	3
Huo	Stage 4	Predominantly cartilage with a small amount of fibrous tissue	4
Huo	Stage 5	Cartilage tissue	5
Huo	Stage 6	Predominantly cartilage with a small amount of immature bone	6
Huo	Stage 7	Equal mixture of cartilage and immature bone	7
Huo	Stage 8	Predominantly immature bone with a small amount of cartilage	8
Huo	Stage 9	Fracture healing with immature bone	9
Huo	Stage 10	Fracture healing with mature bone	10

Lane–Sandhu radiographic criteria are based on references [27, 28], and Huo histological criteria on reference [35]. In the present study, both systems were applied retrospectively to representative archived panels only; the scores were used descriptively and were not treated as animal-level quantitative outcomes.

### Blood and tissue sample collection

On day 35, animals were anesthetized and blood was collected by cardiac puncture into non-heparinized tubes. Samples were allowed to clot for 30 min at room temperature and were centrifuged at 3000 rpm for 15 min at 4 °C. Serum was separated and stored at −80 °C until analysis. Animals were then euthanized by cervical dislocation under deep anesthesia. Femoral and adjacent soft-tissue samples were collected for histological and biochemical assessment.

### Biochemical analyses

### Serum mineral homeostasis and alkaline phosphatase activity

Serum calcium (Ca^2+^; Abcam, catalog no. ab102505) and inorganic phosphate (PO₄^3−^; Abcam, catalog no. ab65622) were measured using commercial colorimetric kits according to the manufacturers’ instructions. Serum magnesium (Mg^2+^) was quantified using a commercial colorimetric magnesium assay kit according to the manufacturer’s instructions. The assay was based on a Mg^2+^-dependent enzyme-linked colorimetric reaction, with absorbance measured at 450 nm; magnesium concentrations were expressed as mg/dL. Serum alkaline phosphatase (ALP) activity was determined using a commercial colorimetric ALP assay kit according to the manufacturer’s instructions. The assay was based on ALP-mediated hydrolysis of p-nitrophenyl phosphate (pNPP) to a colored p-nitrophenol product measured at 405 nm, and ALP activity was expressed as U/L.

### Oxidative-stress markers

Peri-fracture soft tissue was rinsed with ice-cold phosphate-buffered saline (PBS; pH 7.4) and homogenized in ice-cold 50 mM Tris–HCl buffer (pH 7.4) to prepare 10% (w/v) homogenates. Homogenates were centrifuged at 1000 × g for 10 min at 4 °C, and the supernatants were stored at −20 °C until analysis. Malondialdehyde (MDA): lipid peroxidation was assessed as thiobarbituric acid-reactive substances according to Ohkawa et al. (29). Nitric oxide (NO): NO production was estimated indirectly from nitrite accumulation using the Griess method described by Green et al. (30).

### Antioxidant defense indices

*Reduced glutathione (GSH)*: GSH was measured using 5,5’-dithiobis-(2-nitrobenzoic acid) (DTNB). The yellow reaction product was quantified spectrophotometrically at 412 nm (31).

*Superoxide dismutase (SOD)*: SOD activity was determined from inhibition of nitroblue tetrazolium reduction by superoxide generated in the xanthine–xanthine oxidase system and was measured at 560 nm (32).

*Catalase (CAT)*: CAT activity was assessed from hydrogen-peroxide decomposition. Residual H₂O₂ was reacted with 4-aminophenazone and 3,5-dichloro-2-hydroxybenzenesulfonic acid, and the resulting chromophore was quantified at 520 nm (33).

### Inflammatory biomarkers

Inflammatory markers were measured using the commercial ELISA kits, colorimetric assay, or transcription-factor assay described below, according to the manufacturers’ instructions.

C-reactive protein (CRP): rat CRP ELISA kit (Abcam, catalog no. ab256398). Cyclooxygenase-2 (COX-2): COX-2 protein concentration was measured using a commercial ELISA kit suitable for rat samples.

Prostaglandin E₂ (PGE₂): PGE₂ concentration was measured using a competitive ELISA kit (Abcam, catalog no. ab316906).

Myeloperoxidase (MPO): MPO activity, used as an index of neutrophil infiltration, was measured from MPO-catalyzed oxidation of o-dianisidine in the presence of H₂O₂ (34).

Tumor necrosis factor-alpha (TNF-α): rat TNF-α ELISA kit (R&D Systems; catalog no. RTA00).

Interleukin-1 beta (IL-1β): rat IL-1β ELISA kit (Abcam, catalog no. ab255730).

Nuclear factor-kappa B (NF-κB p65): NF-κB p65 DNA-binding activity was assessed using an ELISA-based transcription-factor assay (Abcam, catalog no. ab133112). Colorimetric absorbance was read at 450 nm.

### Bone metabolism and turnover markers

C-terminal telopeptide of type I collagen (CTX-I): serum CTX-I, a marker of type I collagen degradation and bone resorption, was measured using a rat CTX-I ELISA kit (MyBioSource, catalog no. MBS9901663).

Calcitonin: serum calcitonin was measured using a rat calcitonin ELISA kit (Abcam, catalog no. ab289835).

Osteocalcin: serum osteocalcin was measured as a bone-formation-associated marker using a commercial ELISA kit suitable for rat samples.

### Gene expression analysis

Total RNA was extracted from femoral bone tissue using TRIzol reagent (Invitrogen) according to the manufacturer’s protocol. RNA concentration and purity were assessed from the A260/A280 ratio. First-strand cDNA was synthesized from 1 μg total RNA using reverse transcriptase and oligo(dT) primers. Quantitative real-time PCR was performed with SYBR Green master mix to assess the relative mRNA expression of NF-κB, osteoprotegerin (OPG), and bone morphogenetic protein-1 (BMP-1). Three replicate qPCR values per group were available for statistical analysis.

Nuclear factor-kappa B (NF-κB): relative NF-κB mRNA expression was assessed as an inflammatory transcriptional readout in femoral bone tissue.

Osteoprotegerin (OPG): a decoy receptor for RANKL that contributes to regulation of osteoclast differentiation and bone resorption.

Bone morphogenetic protein-1 (BMP-1): an extracellular metalloproteinase involved in procollagen processing and matrix maturation.

Gene expression was normalized to glyceraldehyde-3-phosphate dehydrogenase (GAPDH), and relative expression was calculated using the 2^−ΔΔCt method. Primer sequences used for qRT-PCR are provided in Table 2.

**Table 2:** List of applied primers.

Name	Accession No.	Forward primer	Reverse primer
*Bmp1*	NM_031323.1	CCGACTACACCTACGACCTG	GCCAGTGAAATTCCCTCCGA
*OPG*	NM_012870.2	CACAACCGAGTGTGCGAATG	AAGTGAGCTGCAGTTGGTGT
*NF-κB*	NM_001276711.2	AGACAGCACATAGATGAGCTCCG	GGAAGCCAAATTCGGAAGGC
*GAPDH*	NM_017008.4	AGACAGCCGCATCTTCTTGT	TACGGCCAAATCCGTTCACA

### Histological examination

Femoral specimens were fixed in 10% neutral-buffered formalin for 48 h, decalcified in 10% EDTA for 3 weeks with regular solution changes, and embedded in paraffin. Longitudinal sections (5 μm) were stained with hematoxylin and eosin (H&E) to examine tissue architecture, inflammatory-cell infiltration, callus organization, woven and lamellar bone, and trabecular structure. Representative day-35 fracture micrographs (Figures 2B–D) were retrospectively classified using the 10-point histological fracture-healing scale of Huo et al. (35). The complete histological scoring criteria are summarized in Table 1. Colored arrows in Figure 2 identify the principal morphological features supporting each representative classification. Panel A was intact non-fractured bone and was not scored. Because the complete archived slide set and blinding records were unavailable, these panel-level scores were interpreted descriptively only.

**Figure 2:**
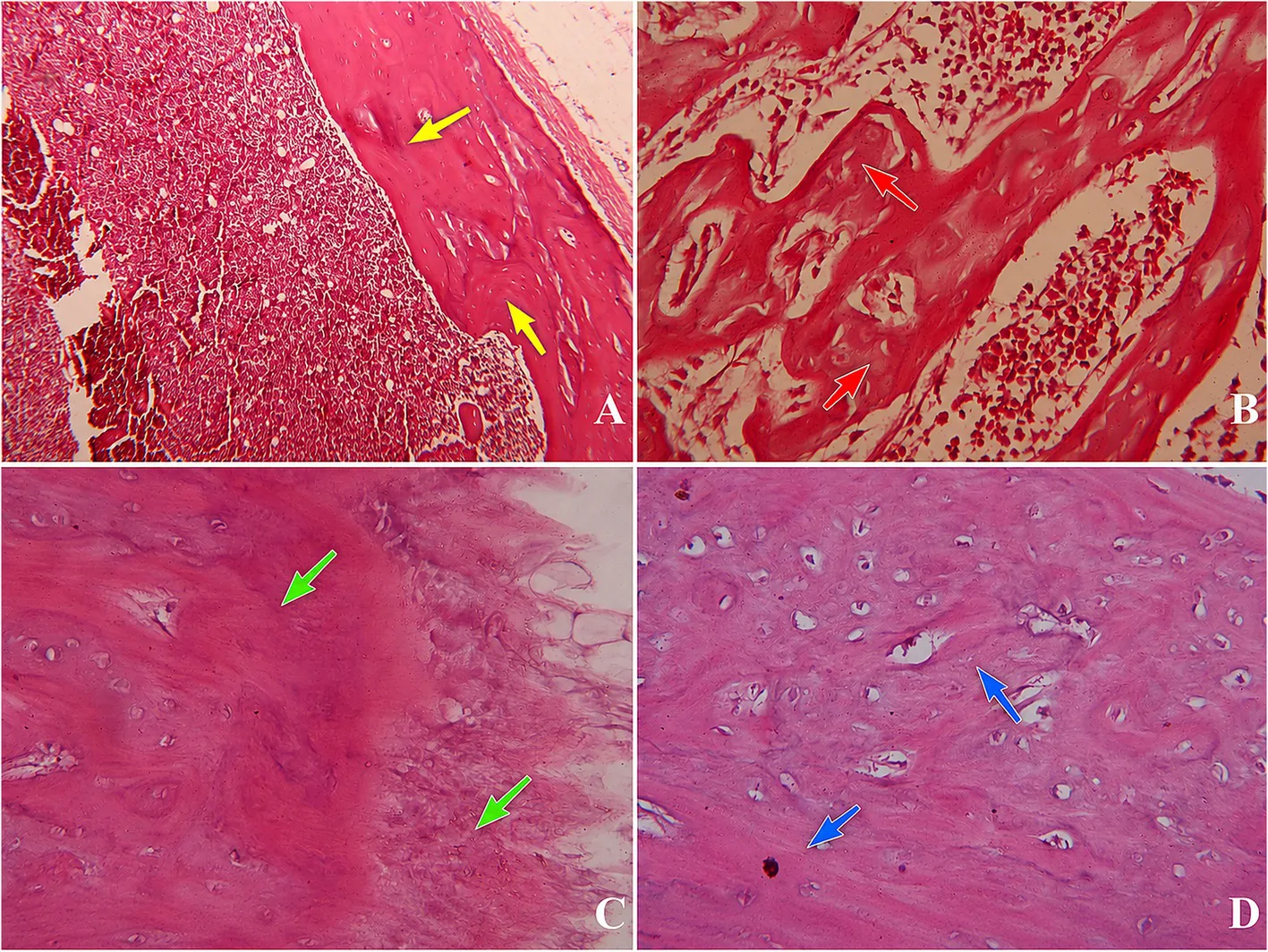
Representative H&E-stained sections used for retrospective descriptive Huo classification. **(A)** Intact control: Organized lamellar cortical bone with osteocyte lacunae (yellow arrows); not scored. **(B)** Fracture control: Predominantly immature woven/trabecular bone with less advanced organization (red arrows), consistent with Huo score 8. **(C)** FX + zingiberene (10 mg/kg/day): Bony union dominated by immature bone (green arrows), consistent with Huo score 9. **(D)** FX + zingiberene-SeNP formulation (0.5 mg/kg/day, total formulation mass): Mature bone with lamellar organization and osteocyte lacunae (blue arrows), consistent with Huo score 10.

### Statistical analysis

Data are expressed as mean ± standard error of the mean (SEM), and numerical grou*p* values for all biochemical and molecular endpoints are provided in Supplementary Table S1. For the biochemical endpoints, six values per group were available for analysis; the qPCR endpoints (NF-κB, OPG, and BMP-1 mRNA) contained three values per group. Each continuous endpoint was analyzed by one-way analysis of variance (ANOVA). To address multiplicity across the complete set of biochemical and molecular endpoints in the analytical dataset, omnibus ANOVA *p* values were adjusted using the Benjamini–Hochberg false discovery rate (FDR) procedure; Bonferroni-adjusted *p* values were also calculated as a conservative secondary multiplicity correction. Pairwise comparisons within each endpoint were performed using Tukey’s multiple-comparison procedure and were interpreted only in the context of an omnibus effect that remained significant after FDR correction. For each endpoint, the direct comparison between the free zingiberene and zingiberene-SeNP groups was specifically examined. The figures report the raw omnibus ANOVA *p* value and the corresponding FDR-adjusted value for each endpoint, while significance brackets denote Tukey-adjusted pairwise comparisons. Statistical significance was defined as adjusted p or q < 0.05; pairwise figure annotations were *p < 0.05, **p < 0.01, ***p < 0.001, and ****p < 0.0001.

## Results

### Available physicochemical characterization of the zingiberene-SeNP formulation

DLS indicated a principal hydrodynamic particle-size range of approximately 400–500 nm, with a PDI of 0.383, indicating a moderately broad particle-size distribution. The measured zeta potential was approximately +10 to +20 mV (Figure 3). These measurements document the available size-distribution, PDI, and surface-charge data but do not establish monodispersity, morphology, loading efficiency, release behavior, stability, cellular uptake, circulation time, or improved bioavailability.

**Figure 3:**
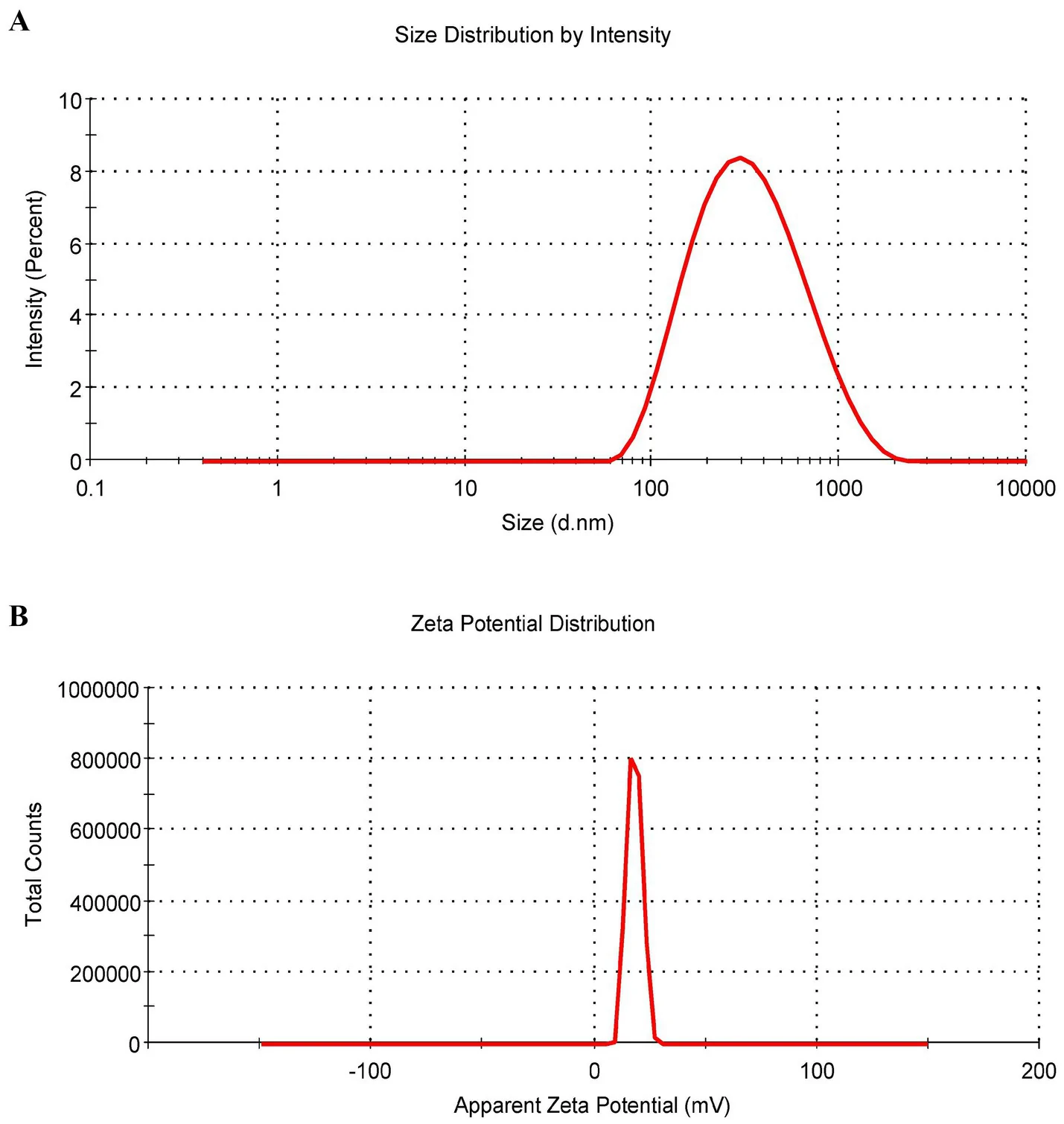
Available characterization of the zingiberene-SeNP formulation: **(A)** hydrodynamic size distribution measured by dynamic light scattering (PDI = 0.383) and **(B)** zeta-potential distribution. These measurements do not provide morphology, loading, release, or stability data.

### Effects on serum mineral homeostasis and ALP activity during fracture healing

Serum calcium, phosphate, magnesium, and ALP activity were assessed as mineral and bone-associated biochemical indices (Figure 4). Relative to the intact control, the fracture-control group had lower calcium (Tukey-adjusted *p* < 0.0001), phosphate (*p* < 0.0001), magnesium (*p* = 0.0247), and ALP activity (*p* < 0.0001). Compared with fracture control, free zingiberene increased calcium (*p* < 0.0001), phosphate (*p* = 0.0002), magnesium (*p* = 0.0247), and ALP activity (*p* < 0.0001). The zingiberene-SeNP formulation increased calcium (*p* = 0.0015), phosphate (*p* = 0.0001), and ALP activity (*p* < 0.0001), whereas the increase in magnesium did not reach statistical significance (*p* = 0.3060). Direct comparison between the two treatment groups showed no significant difference in calcium (*p* = 0.1638), phosphate (*p* = 0.9972), or magnesium (*p* = 0.5478), while ALP activity was significantly higher in the zingiberene-SeNP group than in the free-zingiberene group (*p* = 0.0002). Thus, both treatments improved calcium, phosphate, and ALP relative to fracture control, while the magnesium response was significant only with free zingiberene.

**Figure 4:**
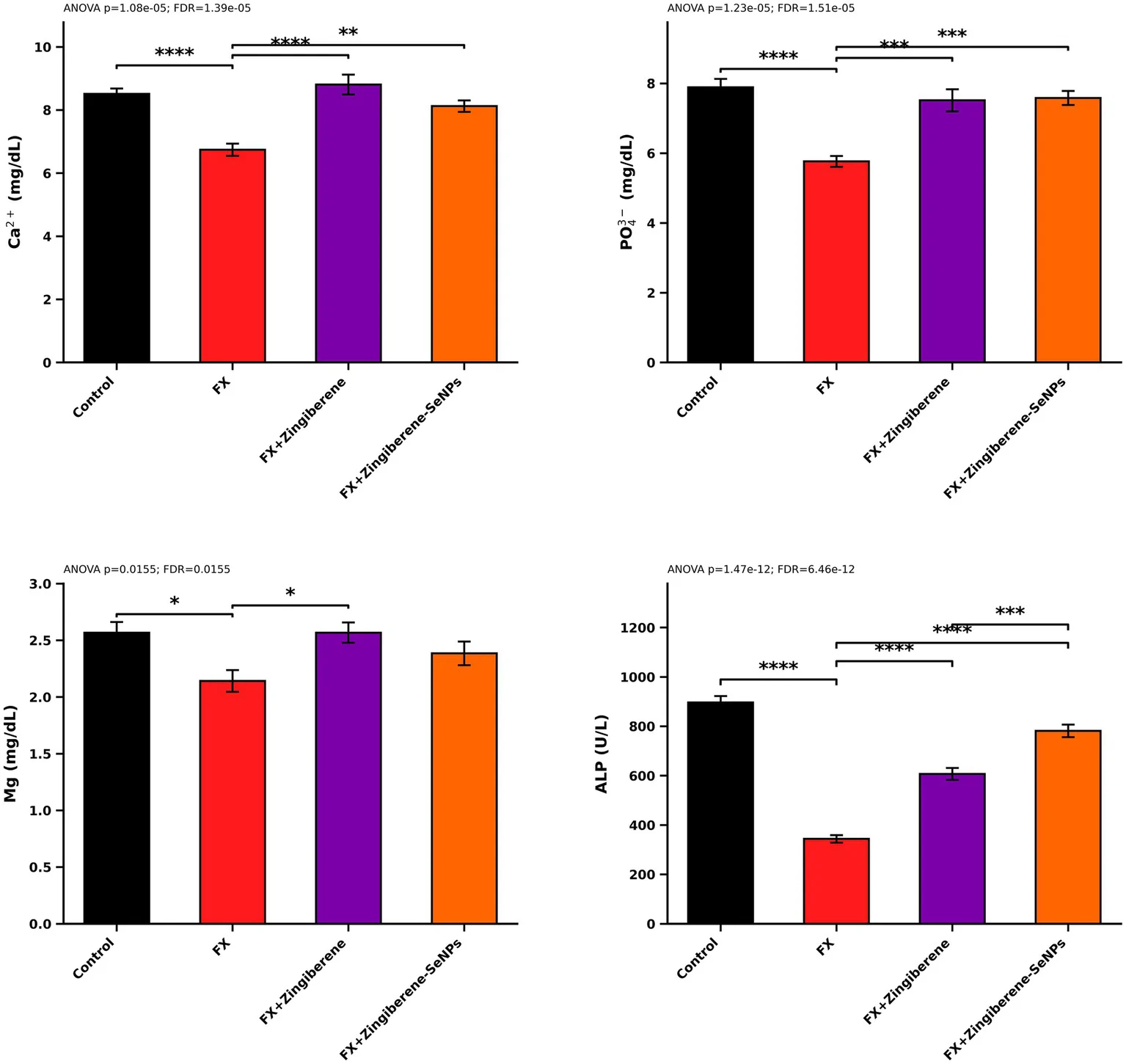
Serum mineral and bone-associated biochemical indices: Calcium (Ca^2+^), inorganic phosphate (PO_4_^3−^), magnesium (Mg^2+^), and alkaline phosphatase (ALP) activity in the experimental groups. Data are mean ± SEM (*n* = 6/group). Control, intact rats; FX, fracture control; FX + zingiberene, fracture treated with zingiberene (10 mg/kg/day); FX + zingiberene-SeNPs, fracture treated with the zingiberene-SeNP formulation (0.5 mg/kg/day, expressed as the total administered formulation mass). Each panel displays the raw one-way ANOVA *p* value and the Benjamini–Hochberg FDR-adjusted value from the global endpoint correction. Brackets indicate Tukey-adjusted pairwise comparisons: *p < 0.05, **p < 0.01, ***p < 0.001, ****p < 0.0001.

### Effects on oxidative stress and antioxidant defense

Oxidative-stress indices and antioxidant defenses were measured in peri-fracture tissue (Figure 5). The fracture-control group had higher MDA and NO than the intact control (both *p* < 0.0001) and lower GSH, SOD, and CAT (all *p* < 0.0001), consistent with marked oxidative disturbance.

**Figure 5:**
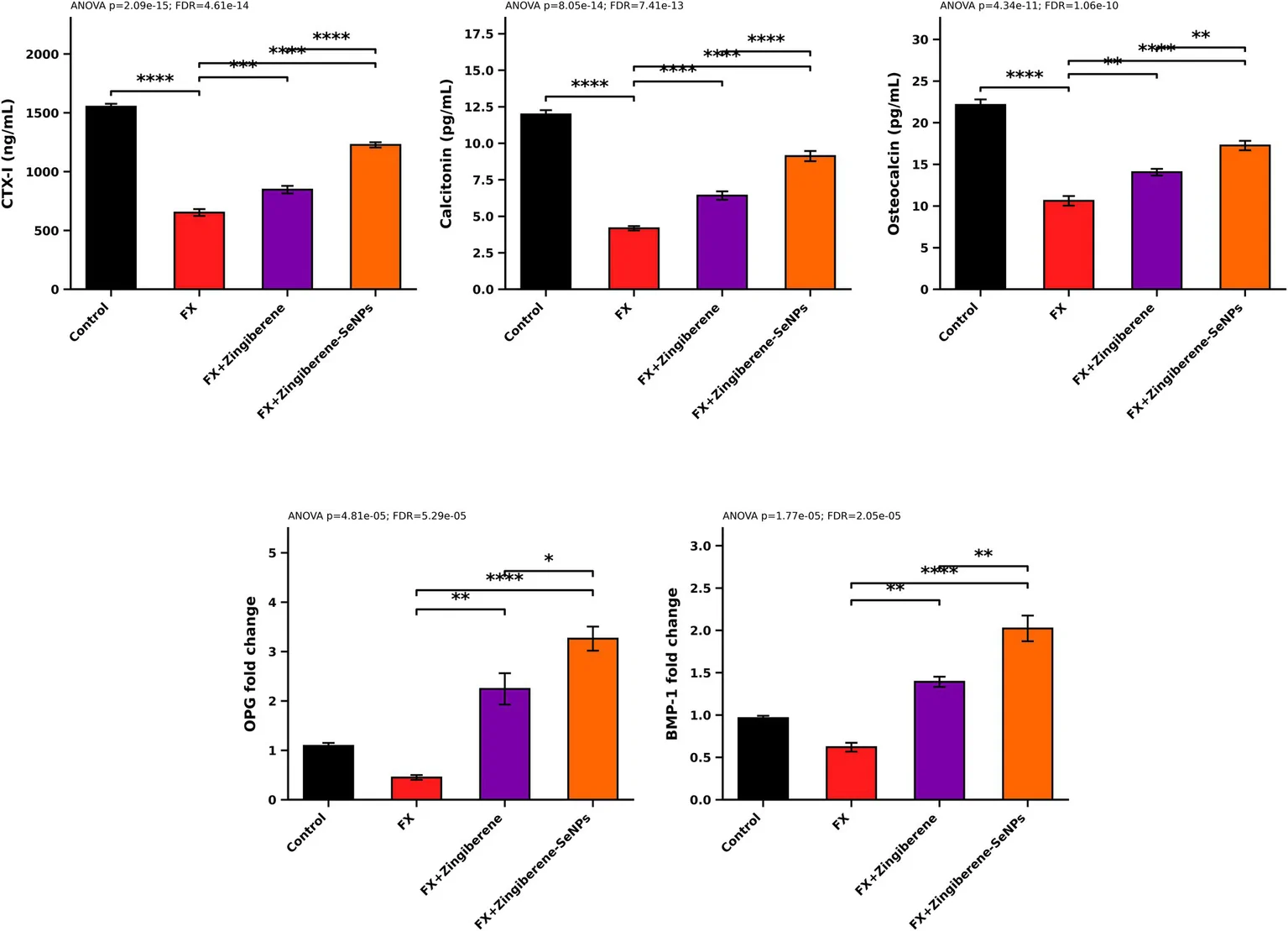
Peri-fracture oxidative-stress and antioxidant indices: malondialdehyde (MDA), nitric oxide (NO), reduced glutathione (GSH), superoxide dismutase (SOD), and catalase (CAT). Data are mean ± SEM (*n* = 6/group). Group abbreviations and doses are as defined in Figure 4. Each panel displays the raw one-way ANOVA *p* value and the Benjamini–Hochberg FDR-adjusted value; brackets indicate Tukey-adjusted pairwise comparisons. *p < 0.05, **p < 0.01, ***p < 0.001, ****p < 0.0001.

Compared with fracture control, free zingiberene reduced MDA (*p* < 0.0001) and NO (*p* < 0.0001). The zingiberene-SeNP formulation likewise reduced MDA and NO (both *p* < 0.0001). Direct treatment-group comparison showed lower MDA with the zingiberene-SeNP formulation than with free zingiberene (*p* = 0.0096), whereas the corresponding difference in NO did not reach significance (*p* = 0.0589).

Fracture was also associated with depletion of endogenous antioxidant defenses. Relative to fracture control, free zingiberene significantly increased SOD (*p* = 0.0045) and CAT (*p* = 0.0003), whereas the increase in GSH was not significant (*p* = 0.1577).

The zingiberene-SeNP formulation significantly increased GSH (*p* < 0.0001), SOD (*p* < 0.0001), and CAT (*p* < 0.0001) relative to fracture control. Direct comparison between treatments showed higher GSH with the zingiberene-SeNP formulation (*p* = 0.0030); SOD (*p* = 0.0536) and CAT (*p* = 0.3316) did not differ significantly between treatments. These findings indicate treatment-associated improvement in antioxidant defense, with statistically supported between-treatment differences limited to selected indices.

### Effects on the inflammatory response in peri-fracture tissue

Inflammatory mediators were assessed in serum or peri-fracture tissue (Figure 6). Compared with the intact control, the fracture-control group showed higher CRP, COX-2, PGE₂, MPO, TNF-α, IL-1β, and NF-κB p65 DNA-binding activity (all Tukey-adjusted *p* < 0.0001).

**Figure 6:**
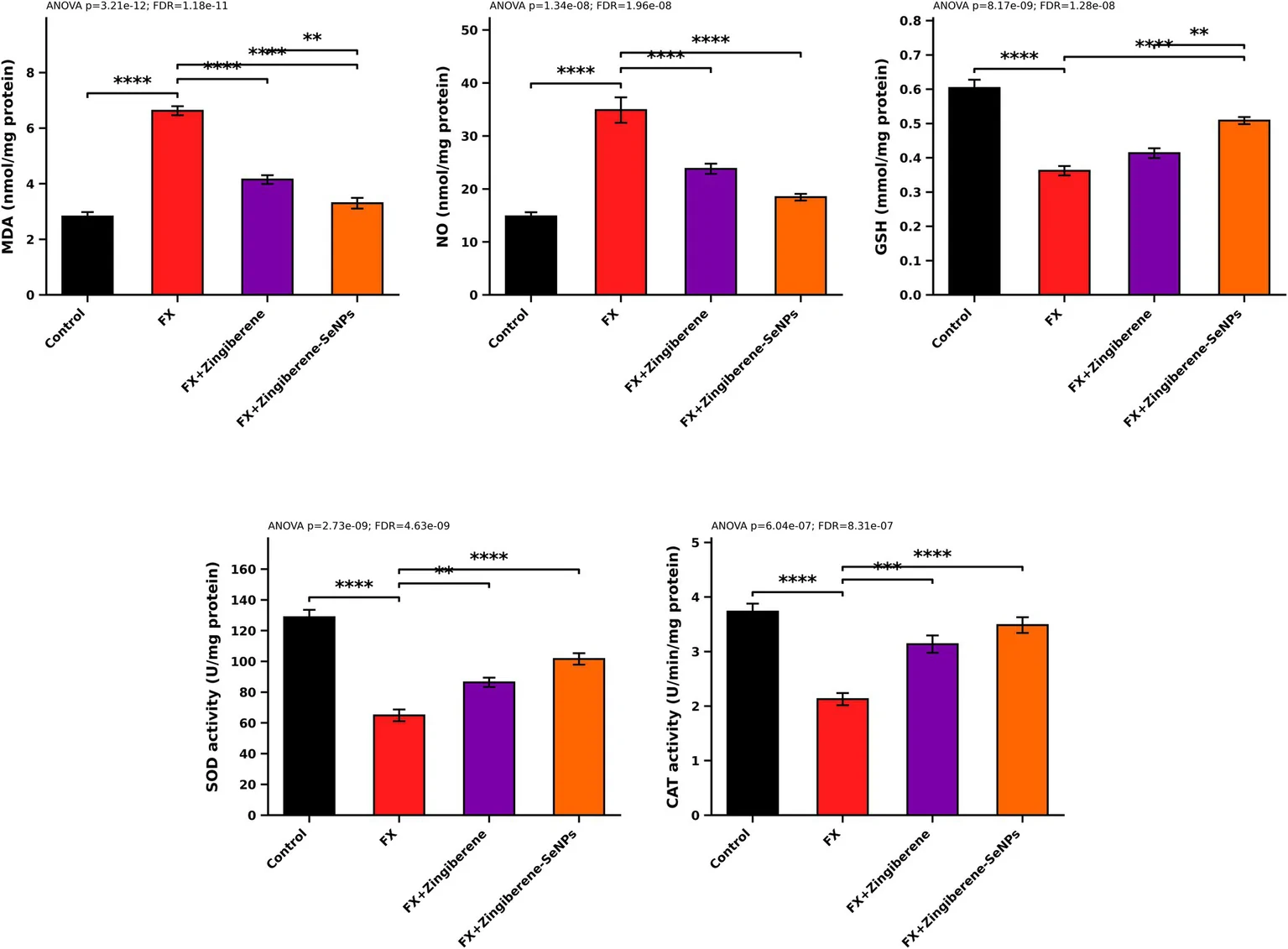
Inflammatory indices: C-reactive protein (CRP), cyclooxygenase-2 (COX-2) concentration, prostaglandin E_2_ (PGE_2_), myeloperoxidase (MPO) activity, tumor necrosis factor-alpha (TNF-α), interleukin-1 beta (IL-1β), NF-κB p65 DNA-binding activity (OD450), and relative NF-κB mRNA expression. Protein/biochemical endpoints are mean ± SEM (*n* = 6/group); NF-κB mRNA is shown as relative fold change from qPCR (*n* = 3/group). Group abbreviations and doses are as defined in Figure 4. Each panel displays the raw one-way ANOVA *p* value and the Benjamini–Hochberg FDR-adjusted value; brackets indicate Tukey-adjusted pairwise comparisons. *p < 0.05, **p < 0.01, ***p < 0.001, ****p < 0.0001.

Compared with fracture control, free zingiberene reduced CRP, COX-2, PGE₂, MPO, IL-1β, and NF-κB p65 (all *p* < 0.0001) and TNF-α (*p* = 0.0001). The zingiberene-SeNP formulation reduced all seven measured inflammatory protein/biochemical endpoints relative to fracture control (all *p* < 0.0001).

Direct comparison of the two treatments showed lower PGE₂ (*p* = 0.0261), TNF-α (*p* < 0.0001), IL-1β (*p* < 0.0001), and NF-κB p65 (*p* < 0.0001) with the zingiberene-SeNP formulation. The treatment groups did not differ significantly for CRP (*p* = 0.0950), COX-2 (*p* = 0.8681), or MPO (*p* = 0.3678).

At the transcript level, NF-κB mRNA expression was higher in fracture control than in intact control (*p* = 0.0004). Both free zingiberene (*p* = 0.0449) and the zingiberene-SeNP formulation (*p* = 0.0017) reduced NF-κB mRNA relative to fracture control; the direct difference between the two treatments was not significant (*p* = 0.1246).

### Effects on bone-turnover markers and selected gene expression

Serum CTX-I, calcitonin, and osteocalcin, together with OPG and BMP-1 mRNA expression, were assessed as selected indices of bone turnover and matrix regulation (Figure 7). The fracture-control group had lower CTX-I, calcitonin, and osteocalcin than the intact control (all *p* < 0.0001). CTX-I is a bone-resorption marker; therefore, its direction of change cannot by itself be interpreted as beneficial or detrimental fracture healing.

**Figure 7:**
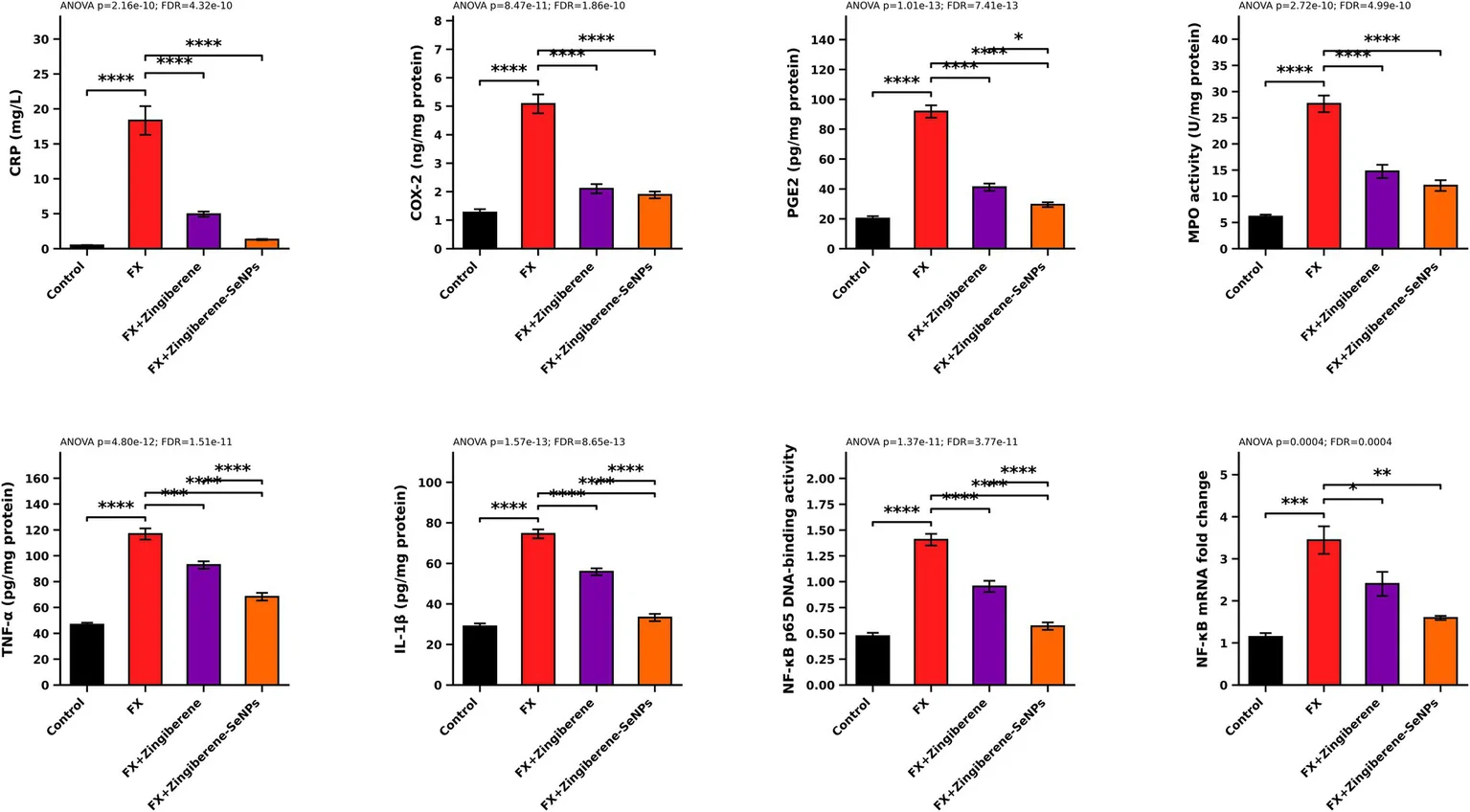
Serum bone-turnover markers (CTX-I, calcitonin, and osteocalcin) and relative OPG and BMP-1 mRNA expression in femoral bone tissue. Serum endpoints are mean ± SEM (*n* = 6/group); qPCR endpoints are relative fold changes (*n* = 3/group). Group abbreviations and doses are as defined in Figure 4. CTX-I is a bone-resorption marker and should not be interpreted in isolation as a beneficial healing endpoint. Each panel displays the raw one-way ANOVA *p* value and the Benjamini–Hochberg FDR-adjusted value; brackets indicate Tukey-adjusted pairwise comparisons. *p < 0.05, **p < 0.01, ***p < 0.001, ****p < 0.0001.

Relative to fracture control, free zingiberene increased CTX-I (*p* = 0.0003), calcitonin (*p* < 0.0001), and osteocalcin (*p* = 0.0017). The zingiberene-SeNP formulation increased all three serum markers relative to fracture control (all *p* < 0.0001). These changes indicate altered bone turnover but do not, by themselves, establish improved mechanical repair.

Direct comparisons showed higher CTX-I (*p* < 0.0001), calcitonin (*p* < 0.0001), and osteocalcin (*p* = 0.0034) in the zingiberene-SeNP group than in the free-zingiberene group. These treatment-group differences support selected biochemical distinctions but not a general conclusion of superior bone anabolism.

ALP activity and serum osteocalcin provided formation-associated biochemical information, whereas CTX-I reflected bone resorption and calcitonin was treated only as a supportive bone-turnover marker. Accordingly, the combined profile was interpreted as altered bone turnover rather than proof of enhanced bone formation.

OPG and BMP-1 mRNA values were numerically lower in fracture control than in intact control, but these pairwise differences were not statistically significant after Tukey adjustment (OPG, *p* = 0.1984; BMP-1, *p* = 0.0886). Relative to fracture control, free zingiberene increased OPG (*p* = 0.0012) and BMP-1 (*p* = 0.0011), while the zingiberene-SeNP formulation increased OPG (*p* < 0.0001) and BMP-1 (*p* < 0.0001). Direct comparison also favored the zingiberene-SeNP formulation for OPG (*p* = 0.0320) and BMP-1 (*p* = 0.0039). These transcripts represent selected regulators of bone remodeling and matrix processing rather than a comprehensive osteogenic pathway panel.

### Radiographic evaluation and representative Lane–Sandhu scores

Figure 8 contains an immediate post-fracture day-0 reference image (panel A), the intact control at day 35 (panel B), and representative day-35 radiographs from the fracture-control (panel C), zingiberene-treated (panel D), and zingiberene-SeNP-treated (panel E) groups. The fracture-control panel showed a persistent fracture line, disorganized callus, and limited remodeling. Retrospective Lane–Sandhu classification according to Table 1 yielded 2 points for callus formation, 2 points for a partial fracture line, and 0 points for remodeling, giving a total score of 4/12. The zingiberene panel showed reduced fracture-line visibility, a more organized callus, and improved bone continuity, although cortical irregularity remained; the corresponding representative score was 7/12 (callus formation 3, fracture line 2, remodeling 2). The zingiberene-SeNP panel showed the least visible fracture line, more extensive mineralized callus, and smoother cortical contours, corresponding to a representative score of 10/12 (callus formation 4, fracture line 4, remodeling 2). Thus, the representative panels showed a descriptive radiographic progression from fracture control to free zingiberene and then to the zingiberene-SeNP formulation. Because these scores were assigned retrospectively to single representative archived panels rather than to blinded replicate radiographs from all animals, they were not statistically analyzed and should not be interpreted as proof of group-level superiority.

**Figure 8:**
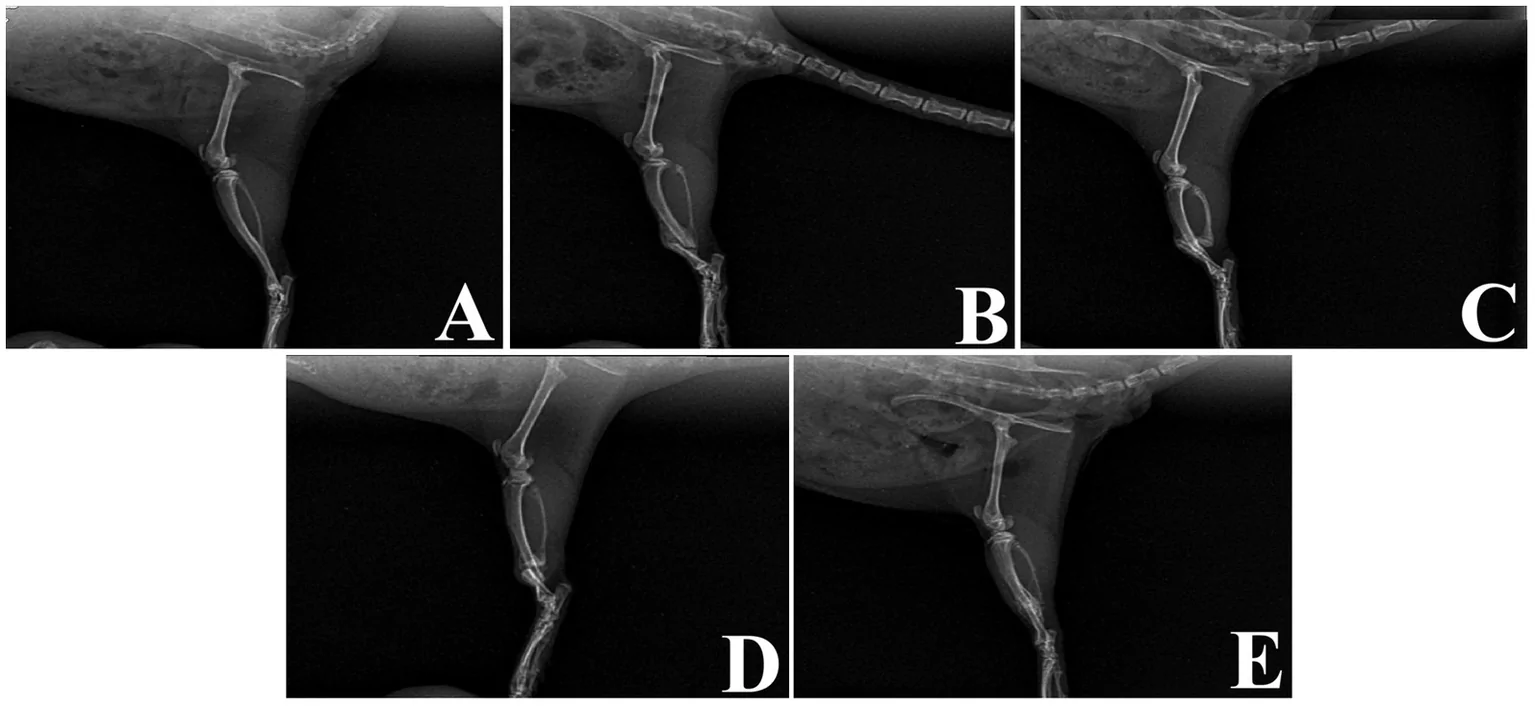
Representative radiographs used for descriptive assessment of fracture healing. **(A)** Immediate post-fracture day-0 reference **(B)** day-35 intact control **(C)** day-35 fracture control **(D)** day-35 FX + zingiberene group (10 mg/kg/day) and **(E)** day-35 FX + zingiberene-SeNP formulation group (0.5 mg/kg/day, total formulation mass).

### Histological evaluation and representative Huo healing scores

H&E-stained sections showed group-dependent differences in callus organization (Figure 2). Intact control bone displayed organized lamellar cortical bone with osteocyte lacunae (panel A; yellow arrows). The fracture-control section showed predominantly immature woven/trabecular bone with less advanced organization (panel B; red arrows), consistent with Huo score 8 (Table 1). The zingiberene-treated section showed bony union dominated by immature bone with improved structural organization (panel C; green arrows), consistent with Huo score 9. The zingiberene-SeNP section showed the most mature appearance, with mature bone displaying lamellar organization and osteocyte lacunae (panel D; blue arrows), consistent with Huo score 10. Thus, the representative panels showed an ordinal progression from score 8 in the untreated fracture control to scores 9 and 10 in the zingiberene and zingiberene-SeNP groups, respectively. These scores were assigned retrospectively to representative archived micrographs rather than to blinded replicate sections from all animals; consequently, they were not statistically analyzed and should be interpreted as semi-quantitative morphological support rather than proof of group-level statistical superiority.

## Discussion

This exploratory study examined free zingiberene and a zingiberene-SeNP formulation in a rat femoral fracture model. Both interventions were associated with improvements in multiple mineral, oxidative, inflammatory, bone-turnover, and molecular endpoints relative to fracture control. Direct treatment-group comparisons favored the nanoformulation for selected endpoints, including MDA, GSH, ALP, PGE₂, TNF-α, IL-1β, NF-κB p65, CTX-I, calcitonin, osteocalcin, OPG, and BMP-1, but not for every outcome. Therefore, the data do not establish global superiority, synergy, or a formulation-specific mechanism. The representative-panel Huo assessment was consistent with progressive histological maturation (scores 8, 9, and 10 in the fracture-control, zingiberene, and zingiberene-SeNP panels, respectively).

Fracture was associated with lower serum calcium, phosphate, magnesium, and ALP activity. Both treatments increased calcium and phosphate relative to fracture control, whereas the magnesium response was more modest: free zingiberene significantly increased magnesium, while the increase with the zingiberene-SeNP formulation was not significant. ALP activity increased significantly with both treatments and was higher with the zingiberene-SeNP formulation than with free zingiberene. Calcium, phosphate, and magnesium contribute to mineral homeostasis and skeletal mineralization (36, 37), while ALP is a bone-formation-associated biochemical marker. Nevertheless, serum mineral concentrations and ALP activity alone do not demonstrate mineral incorporation into the fracture callus, improved bone microarchitecture, or greater mechanical strength; these findings should therefore be interpreted as supportive biochemical changes rather than direct evidence of functional bone restoration.

Both treatments reduced CRP, COX-2, and PGE₂ concentrations, MPO activity, TNF-α, IL-1β, and NF-κB p65 DNA-binding activity relative to fracture control, and both also reduced NF-κB mRNA expression in the qPCR dataset. Previous studies have linked ginger-derived compounds to modulation of RANKL, Akt, NF-κB, and PGE₂ signaling (20, 21, 38, 39). In the present study, however, Akt and RANKL signaling were not directly measured. Direct comparisons favored the zingiberene-SeNP formulation for PGE₂, TNF-α, IL-1β, and NF-κB p65 DNA-binding activity, whereas CRP, COX-2 concentration, MPO activity, and NF-κB mRNA did not differ significantly between treatments. These results therefore support selected, rather than uniform, between-treatment differences in inflammatory regulation.

Fracture was associated with increased MDA and NO and reduced GSH, SOD, and CAT. Free zingiberene significantly reduced MDA and NO and increased SOD and CAT, although its increase in GSH was not significant after Tukey adjustment. The zingiberene-SeNP formulation significantly improved all five indices relative to fracture control and showed lower MDA and higher GSH than free zingiberene; the direct differences for NO, SOD, and CAT were not significant. Selenium is incorporated into antioxidant selenoproteins, and selenium-containing nanoparticles have been reported to affect redox regulation (10, 40, 41). Nevertheless, glutathione peroxidase activity, selenium status, and selenoprotein expression were not measured here, so a selenium-specific antioxidant mechanism cannot be concluded.

Prior studies have associated selenium nanoparticles with JNK/FOXO3-related redox regulation and with osteogenic differentiation (40, 41). These pathways provide biological context but were not interrogated in the present experiment. The current data are therefore correlative and indicate changes in measured biomarkers rather than activation of a defined molecular pathway. Establishing causality will require pathway-specific perturbation experiments, such as pharmacological inhibition or genetic manipulation, together with protein-level and downstream signaling validation.

The changes in CTX-I, calcitonin, osteocalcin, OPG, and BMP-1 indicate altered bone turnover and matrix regulation. Direct comparisons favored the zingiberene-SeNP formulation over free zingiberene for all five of these endpoints. However, OPG and BMP-1 did not differ significantly between intact and fracture control after Tukey adjustment, and the qPCR analyses were based on three values per group. OPG participates in RANKL-dependent control of osteoclast differentiation, while BMP-1 contributes to extracellular-matrix processing (42, 43). CTX-I reflects type I collagen degradation and bone resorption (44, 45); an increase during healing is not inherently beneficial and must be interpreted in the context of formation markers, structural remodeling, and mechanical function. Calcitonin is also not a core stand-alone fracture-healing endpoint (46–48). Consequently, these markers should be considered supportive rather than definitive evidence of enhanced osteogenesis.

Evidence that 6-gingerol can influence osteoblast differentiation and mineralized matrix formation (49, 50) is relevant to ginger pharmacology but cannot be directly extrapolated to purified zingiberene. Similarly, reports that selenium nanoparticles activate BMP-2/MAPK/β-catenin signaling (51, 52) do not demonstrate that this pathway mediated the effects observed here. Accordingly, these literature-derived pathways are presented only as hypotheses providing biological context and should not be interpreted as mechanisms demonstrated by the present study.

The molecular assessment was limited to NF-κB, OPG, and BMP-1 mRNA, with three qPCR values per group. Although ALP activity and serum osteocalcin protein were measured, the study did not include a broader formation and resorption panel such as RUNX2, COL1A1, osteocalcin protein in callus tissue, TRAP, RANKL, or the RANKL/OPG ratio. This restricted molecular panel and small qPCR sample size limit mechanistic inference and prevent a detailed distinction between bone formation and resorption.

H&E histology and radiography were consistent with improved callus organization in both treatment groups, with the most advanced appearance in the zingiberene-SeNP group. Retrospective application of the Lane–Sandhu radiographic score (27, 28) (Table 1) to the representative day-35 fracture panels yielded scores of 4/12, 7/12, and 10/12 for the fracture-control, zingiberene, and zingiberene-SeNP panels, respectively. Similarly, application of the Huo histological healing scale (35) (Table 1) to the representative day-35 fracture micrographs classified the corresponding fracture-control, zingiberene, and zingiberene-SeNP panels as scores 8, 9, and 10, respectively. Together, these representative-panel classifications support a morphological progression toward more advanced callus organization and maturation with treatment. However, the available records did not permit blinded scoring of replicate radiographs or histological sections from all animals, and histomorphometry, micro-CT, and biomechanical testing were unavailable. Accordingly, both the Lane–Sandhu and Huo values should be interpreted as descriptive semi-quantitative classifications rather than statistically validated group-level endpoints.

Nanoparticle formulations can, in principle, modify cellular uptake, stability, release, and tissue distribution (8, 9, 12, 53). In this study, however, pharmacokinetics, tissue distribution, loading efficiency, release kinetics, and systemic selenium exposure were not measured. In addition, the free-zingiberene and formulation doses were not equivalent, and an SeNP-only group was absent. Improved bioavailability, targeted delivery, selenium-specific activity, and synergy are therefore plausible explanations but were not demonstrated.

Several limitations qualify these findings. The group size (n = 6/group) was selected based on comparable published animal studies rather than a formal *a priori* power calculation, and the qPCR analyses were based on three values per group. Physicochemical characterization was limited to DLS-derived size distributionand PDI, together with zeta-potential analysis. The unfixed fracture model with unrestricted activity may have introduced variability, and fracture alignment was not quantified using a standardized alignment score. The 0.5 mg/kg/day dose represented the total administered formulation mass; however, the individual selenium and zingiberene contents within this dose were not separately quantified. No biomechanical testing, comprehensive osteogenic/resorption panel, pharmacokinetic analysis, blood selenium measurement, body-weight trajectory, liver or kidney assessment, major-organ histology, immune-toxicity evaluation, or long-term accumulation study was performed. Accordingly, the study cannot directly determine whether the observed radiographic, histological, and biochemical improvements translated into greater mechanical strength or functional integrity of the healed femur. Finally, the rat model limits direct clinical extrapolation. The findings should therefore be regarded as hypothesis-generating and require independent confirmation. Accordingly, the present findings should not be interpreted as evidence of systemic safety or long-term therapeutic suitability of the zingiberene-SeNP formulation. Comprehensive toxicological, pharmacokinetic, biodistribution, and long-term safety studies are required before translational therapeutic conclusions can be made.

## Conclusion

Free zingiberene and the zingiberene-SeNP formulation produced favorable changes in the biochemical and molecular environment associated with femoral fracture repair. Both treatments attenuated oxidative and inflammatory disturbances, improved selected mineral-homeostasis and bone-associated biochemical indices, and modulated selected bone-turnover and matrix-regulatory markers. The zingiberene-SeNP formulation showed stronger responses than free zingiberene for several selected endpoints, while the radiographic and representative histological findings were consistent with more advanced callus organization and bone maturation. However, these findings represent preliminary preclinical evidence and do not establish systemic safety or long-term therapeutic suitability. Further studies incorporating dose-equivalent controls, comprehensive nanoparticle characterization, quantitative imaging, biomechanical testing, pharmacokinetic and biodistribution analyses, circulating selenium measurements, and systemic toxicity assessment are required before the translational potential of the zingiberene-SeNP formulation can be established and its underlying molecular mechanisms clarified.

## Data Availability

The original contributions presented in the study are included in the article/Supplementary material, further inquiries can be directed to the corresponding author/s.
