# Supplementary material for: A zingiberene–selenium nanoparticle formulation modulates oxidative, inflammatory, and osteogenic responses during femoral fracture healing in rats
Source: Front Vet Sci. 2026 Oct 2;13:1908406. doi: 10.3389/fvets.2026.1908406 (PMC13672958; doi:10.3389/fvets.2026.1908406)
Supplement: Supplementary file 1 [file Table_1.DOCX]

**Supplementary Table S1. Mean ± SEM values of all measured biochemical and molecular parameters across experimental groups**

Values are presented as mean ± SEM.

| **Parameter** | **Control** | **FX** | **FX + zingiberene** | **FX + zingiberene-SeNPs** | **ANOVA p** |
| --- | --- | --- | --- | --- | --- |
| Calcium | 8.51 ± 0.17 | 6.74 ± 0.20 | 8.81 ± 0.32 | 8.12 ± 0.18 | <0.0001 |
| Phosphate | 7.89 ± 0.24 | 5.77 ± 0.16 | 7.52 ± 0.32 | 7.58 ± 0.20 | <0.0001 |
| Magnesium | 2.57 ± 0.09 | 2.14 ± 0.10 | 2.57 ± 0.09 | 2.39 ± 0.10 | 0.0155 |
| Alkaline phosphatase (ALP) | 896.82 ± 25.77 | 344.06 ± 15.24 | 607.05 ± 24.10 | 781.69 ± 25.43 | <0.0001 |
| Malondialdehyde (MDA) | 2.82 ± 0.16 | 6.62 ± 0.16 | 4.15 ± 0.16 | 3.30 ± 0.19 | <0.0001 |
| Nitric oxide (NO) | 14.82 ± 0.78 | 34.88 ± 2.41 | 23.79 ± 0.97 | 18.44 ± 0.63 | <0.0001 |
| Reduced glutathione (GSH) | 0.604 ± 0.024 | 0.362 ± 0.014 | 0.413 ± 0.014 | 0.509 ± 0.010 | <0.0001 |
| Superoxide dismutase (SOD) | 128.68 ± 4.82 | 64.91 ± 3.78 | 86.37 ± 3.03 | 101.56 ± 3.71 | <0.0001 |
| Catalase (CAT) | 3.73 ± 0.15 | 2.13 ± 0.11 | 3.14 ± 0.16 | 3.48 ± 0.14 | <0.0001 |
| C-reactive protein (CRP) | 0.47 ± 0.05 | 18.34 ± 2.05 | 4.94 ± 0.37 | 1.30 ± 0.07 | <0.0001 |
| Cyclooxygenase-2 (COX-2) | 1.27 ± 0.12 | 5.08 ± 0.33 | 2.11 ± 0.16 | 1.89 ± 0.12 | <0.0001 |
| Prostaglandin E₂ (PGE₂) | 20.11 ± 1.63 | 91.87 ± 4.12 | 41.17 ± 2.48 | 29.46 ± 1.58 | <0.0001 |
| Myeloperoxidase (MPO) | 6.12 ± 0.37 | 27.65 ± 1.59 | 14.76 ± 1.27 | 12.04 ± 1.03 | <0.0001 |
| Tumour necrosis factor-α (TNF-α) | 46.63 ± 1.58 | 116.87 ± 4.33 | 92.84 ± 2.89 | 68.30 ± 2.99 | <0.0001 |
| Interleukin-1β (IL-1β) | 28.97 ± 1.42 | 74.56 ± 2.19 | 55.88 ± 1.71 | 33.28 ± 1.80 | <0.0001 |
| Nuclear factor-κB (NF-κB) | 0.473 ± 0.032 | 1.407 ± 0.056 | 0.955 ± 0.054 | 0.570 ± 0.036 | <0.0001 |
| NF-κB mRNA expression | 1.14 ± 0.09 | 3.44 ± 0.33 | 2.40 ± 0.29 | 1.59 ± 0.05 | 0.0004 |
| C-terminal telopeptide of type I collagen (CTX-I) | 1552.18 ± 24.33 | 652.60 ± 29.02 | 847.59 ± 32.23 | 1227.66 ± 22.23 | <0.0001 |
| Calcitonin | 11.98 ± 0.29 | 4.18 ± 0.15 | 6.42 ± 0.29 | 9.13 ± 0.35 | <0.0001 |
| Osteocalcin | 22.13 ± 0.68 | 10.62 ± 0.57 | 14.06 ± 0.40 | 17.27 ± 0.57 | <0.0001 |
| OPG mRNA expression | 1.09 ± 0.06 | 0.45 ± 0.05 | 2.24 ± 0.32 | 3.26 ± 0.24 | <0.0001 |
| BMP-1 mRNA expression | 0.96 ± 0.03 | 0.62 ± 0.05 | 1.39 ± 0.06 | 2.02 ± 0.15 | <0.0001 |

Abbreviations: FX, fracture control; SeNPs, selenium nanoparticles.
